# Architecture of SWI/SNF chromatin remodeling complex

**DOI:** 10.1007/s13238-018-0524-9

**Published:** 2018-03-15

**Authors:** Zhihui Zhang, Xuejuan Wang, Jiyu Xin, Zhenrui Ding, Sheng Liu, Qianglin Fang, Na Yang, Rui-min Xu, Gang Cai

**Affiliations:** 1 Hefei National Laboratory for Physical Sciences at Microscale and School of Life Sciences, University of Science and Technology of China, Hefei, 230027 China; 2 CAS Center for Excellence in Molecular Cell Science, Chinese Academy of Sciences, Hefei, 230026 China; 3 National Laboratory of Biomacromolecules, Institute of Biophysics, Chinese Academy of Sciences, Beijing, 100101 China

**Dear Editor,**

The SWI/SNF complex is a large ATP-dependent chromatin remodeling complex that is highly conserved from yeast to human, which is essential for transcription regulation, genomic stability, DNA repair and many aspects of development (Kasten et al., 2011). SWI/SNF contains more than 10 subunits (M.W. >1 MDa), including a DNA-dependent ATPase subunit and several accessory subunits. Mutations in several SWI/SNF subunits have been recently identified at a high frequency in a variety of cancers (Masliah-Planchon et al., 2015). Despite the fundamental importance, the structural information of the SWI/SNF complex has been limited to only low-resolution electron microscopy (EM) reconstructions (23–30 Å) without subunits localization and the lack of SWI/SNF-nucleosome structure (Smith et al., 2003; Dechassa et al., 2008).

We aimed to determine the improved 3D EM reconstruction of intact SWI/SNF complex and to illuminate the subunit architecture (Fig. 1A). Firstly, we optimized the affinity purification steps and obtained highly homogeneous samples of SWI/SNF endogenously purified from *S*. *cerevisiae* (Figs. 1B and S1). The negative-stain EM analysis suggested SWI/SNF resembles like a lion, harboring foreleg, thigh, tail, head and belly regions (Fig. 1C) that adopts three major conformations differing in the position of the thigh domain (Fig. 1B).

**Figure 1:**
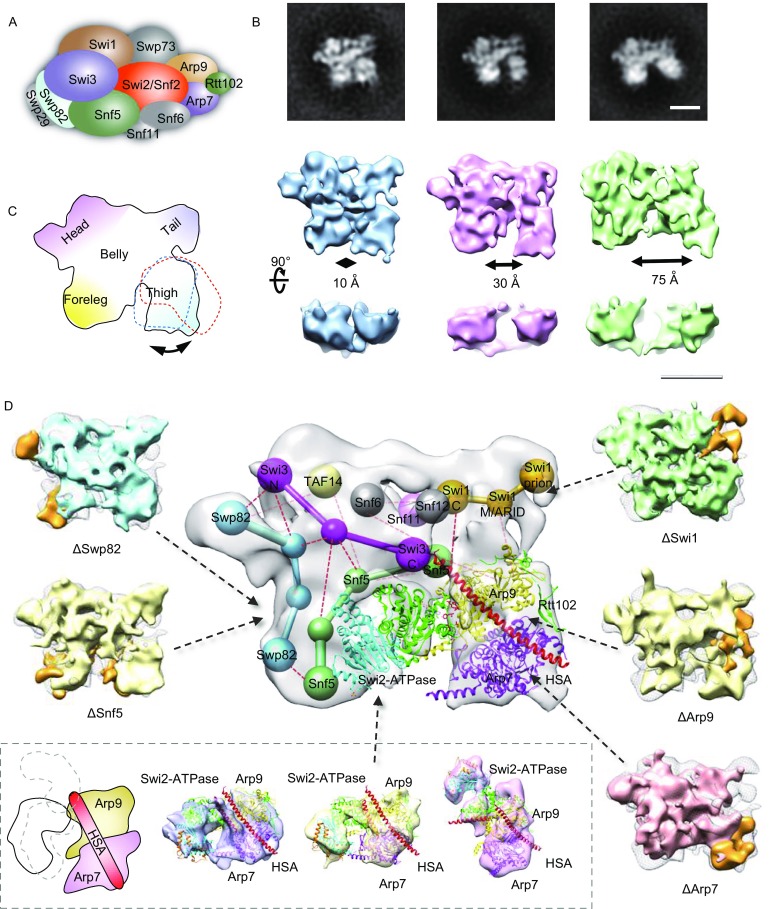
**Architecture of the SWI/SNF complex**. (A) A schematic view of the subunit composition of the yeast SWI/SNF. (B) 2D and 3D EM analysis of SWI/SNF complex showing variability in the distance of the foreleg and thigh. Three different conformations were identified through reference-free alignment and classification of EM images. Scale bar, 100 Å. (C) A schematic view of SWI/SNF structure is shown to highlight the substantial mobility of the thigh module. (D) Identification of Swp82 and Snf5 (left panel), Swi1, Arp7 and Arp9 (right panel) subunits through 3D difference mapping (solid orange density) by comparing the structure of each subunit deletion mutant (cyan-ΔSwp82, yellow-ΔSnf5, green-ΔSwi1, yellow-ΔArp9, hot pink-ΔArp7) with that of an intact SWI/SNF (gray mesh). EM structures and schematic of the catalytic core sub-complex containing Snf2, Arp7/9 and Rtt102 that are fitted by the crystal structure of Snf2 HSA-Arp7-Arp9 and Rtt102 (PDB ID: 4I6M) (Schubert et al., 2013) and Snf2 post-HSA-ATPase-SnAc domains (PDBID: 5HZR) (Xia et al., 2016) highlighting the substantial mobility of the ATPase domain (bottom panel). The structural schematic of the SWI/SNF complex showing the subunit architecture (middle panel). The information is determined through combination of the structural architecture and subunit interaction information derived from the CX-MS analysis (Sen et al., 2017). The crystal structure of Snf2 HSA-Arp7-Arp9 and Rtt102 (PDB ID: 4I6M) and Snf2 post-HSA-ATPase-SnAc domains (PDBID: 5HZR) are fitted in the EM density as rigid bodies. The Snf2 ATPase domain directly interacts with Snf5 in the foreleg and with the ARP module in thigh region

We next delineated the modular architecture of SWI/SNF by deleting different subunits once at a time from the complex and comparing the structure of intact SWI/SNF with those missing particular subunits. The Snf2-Arp7-Arp9 ternary complex is the catalytic core of SWI/SNF and harbors more than 90% of the catalytic activity of intact SWI/SNF (Yang et al., 2007). Therefore, we firstly focused on the location of the Arp7 and Arp9 subunits by 3D reconstruction of the Arp7 or Arp9 deletion mutants (Figs. S2 and S3; Table S1). Through detailed image classification, we found Arp7 and Arp9 are the primary constituents of the thigh region (Fig. 1D, right panel), which impart an important regulatory function on the Snf2 ATPase activity (Szerlong et al., 2008; Schubert et al., 2013).

To pinpoint the location of the Snf2 ATPase in the intact holo-SWI/SNF structure, we acquired the endogenous Snf2-Arp7-Arp9 catalytic core and determined its 3D structure (Fig. S4). The 3D structure we obtained was unambiguously fitted by the available crystal structures of Snf2 HSA-Arp7-Arp9 (PDBID: 4I6M) (Schubert et al., 2013) and Snf2 post-HSA-ATPase-SnAc domains (PDBID: 5HZR) (Xia et al., 2016) (Fig. 1D, bottom panel). The Arp module including Arp7, Arp9 and the Snf2 HSA domain are relatively stable in the Snf2-Arp7-Arp9 assembly, whereas the pHSA-ATPase-SnAc domains undergo a large range of motion. This observation suggests that the ATPase is highly dynamic, which is consistent with the conformational regulation of the ATPase domain for chromatin remodeling (Xia et al., 2016; Liu et al., 2017).

Furthermore, we examined the locations of the Snf5, Swp82 subunits, which contact with the open histone octamer face of the nucleosomes (Dechassa et al., 2008) and of the Swi1 subunit, which coupled with Snf5 are responsible for binding to transcription factors (Prochasson et al., 2003). By subunit deletion analysis (Table S1), we identified Snf5 (Fig. S6) and Swp82 (Fig. S5) overlap in the foreleg region of SWI/SNF (Fig. 1D, left panel). A portion of Swp82 is located in the head region and part of the Snf5 appears to form a bridge between the foreleg and thigh regions. Structural analysis of the ∆Swi1 mutant suggests Swi1 (Fig. S7) is localized in the tail region that leans above the ARP module (Fig. 1D, right panel). A significant portion of Swi1 is distal to Snf5, even though they associate together at the belly region as also seen by CX-MS (Sen et al., 2017). The peripheral location of the Swi1 and Snf5 are consistent with the *in vivo* functions to bind to transcription factors (Prochasson et al., 2003).

Remarkably, the mobility of the thigh region corresponding to the Snf2 HSA-Arp7-Arp9 catalytic core is substantially increased in the ∆Snf5 complex compared to the wild type (Fig. S6C). The Snf2-Arp7-Arp9 could even be fully detached in the absence of Snf5, highlighting the key role of Snf5 in coordinating the distinct modules of SWI/SNF (Dutta et al., 2017; Sen et al., 2017). We also observe the dissociation of the catalytic core in the ∆Swp82 mutant (Fig. S5C). These observations suggest that the stability of the catalytic core highly depends on the foreleg region, especially the Snf5’s contribution.

We combined the structural information gathered by our EM studies with the subunit interaction information acquired from the CX-MS (Sen et al., 2017). The unprecedented details of the subunit architecture of the whole SWI/SNF complex are revealed (Fig. 1D, middle panel). A number of crosslinks that Snf5 associate with the ATPase domain of Snf2 and with the Swp82, consistent with the presence of Snf5 and Swp82 in the foreleg region. Snf5 is also frequently cross-linked to the Swi3 and Swi1 subunits and slightly less to Snf6, consistent with its potential role as a bridge between Snf2 and the rest of the SWI/SNF complex. Consistent with the vital role of Snf5 in the enzymatic activity of SWI/SNF (Sen et al., 2017), the Snf5 interacts directly with the ATPase domain of Snf2 (Fig. 1D, middle panel). The two lobes of the ATPase domain are anchored to the two legs, with lobe2 contacting the Snf5 subunit in the foreleg and with lobe1 binding to the ARP module in the thigh region. Our analysis reveals subunit architecture of the SWI/SNF complex and highlights functional significance of Snf5 subunit and Arp module grabbing the two lobes of the Snf2 ATPase domain.

A major challenge in understanding mechanisms of chromatin remodeling has been the lack of structural insight into how SWI/SNF engages nucleosomes. We found that SWI/SNF forms a stable complex with nucleosomes in the absence of ATP (Figs. 2A, 2C and S8). The recently published cryo-EM reconstruction of the Snf2-nucleosome assembly (PDBID: 5X0Y) (Liu et al., 2017) could be unambiguously fitted as a rigid body into the SWI/SNF-nucleosome structure (Fig. 2B, state I). The density of the nucleosomal substrate is gripped in a pincer-like fashion by the Snf5 and Snf2 ATPase in the foreleg and the ARP module in the thigh (Fig. 2B). The observed SWI/SNF nucleosome binding mode is highly distinct from the prevailing model in which nucleosomes are either bound in a cleft of SWI/SNF (Dechassa et al., 2008) or are caged by RSC (Chaban et al., 2008). The locations of the Snf5, Swp82 and Snf2 subunits and the observed nucleosome-binding mode are consistent with previous SWI/SNF-nucleosome cross-linking data (Dechassa et al., 2008). The two lobes of the Snf2 ATPase domain spanning the two legs of the SWI/SNF complex make the most extensive contacts with both the nucleosome DNA and histone surface, which is consistent with previous functional data showing that Snf2 provides most of the molecular contacts with nucleosomes and Snf5/Swp82 interact extensively on the same side of the histone surface (Dechassa et al., 2008; Liu et al., 2017).

**Figure 2:**
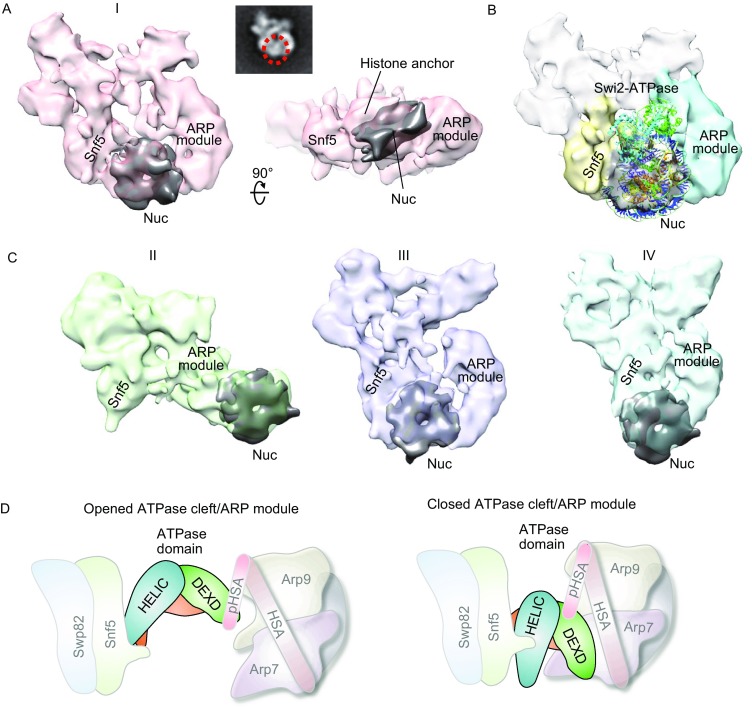
**3D reconstructions of SWI/SNF-nucleosome assembly in different states**. (A) 2D and 3D structure of typical SWI/SNF-nucleosome assembly (state I). The dark gray density corresponds to a nucleosome. (B) Details of the SWI/SNF-nucleosome interfaces. The recently published cryo-EM reconstruction of the Snf2-nucleosome assembly (PDBID: 5X0Y) (Liu et al., 2017) is fitted as a rigid body into the SWI/SNF-nucleosome structure. (C) EM reconstructions of the other three intermediate states of SWI/SNF stably binding to a nucleosome (state II, III and IV). (D) Schematic diagram of the structural rearrangements of the Arp module during nucleosome binding. The mobility of the Arp module may modulate the conformation of the ATPase domain as a conformational switch on the SWI/SNF remodeling activity

In another binding mode, the nucleosomal substrate is tethered only to the tip of the ARP module (Fig. 2C, state II). This observation suggests that the ATPase domain is not required for stable nucleosome binding and the ARP module alone can initiate nucleosome binding. Consistent with the ARPs functions in INO80 complex (Shen et al., 2003), the SWI/SNF sub-complex, containing Arp7-Arp9-Snf2^HSA^-Rtt102, shifts both free DNA and 174-bp nucleosomes, suggesting that the ARP module in SWI/SNF substantially contributes to the nucleosome binding and the primary interaction is made through extra-nucleosomal DNA. Given that the Arp module is highly conserved, and is found in many chromatin-modifying complexes, it can be argued that the Arp module could be universally used by chromatin-remodeling complexes to contact linker or nucleosomal DNA and initiate nucleosome binding. Through the continuous conformational changes of the ARP module, the nucleosome substrate could be engaged on the ATPase catalytic core and promote concerted nucleosomal binding.

Although the ARP module is not required for remodeler assembly of SWI/SNF or RSC complex, it impart an important regulatory function on the ATPase activity (Szerlong et al., 2008; Schubert et al., 2013). Snf5 subunit plays a critical role in modulating the ARP mobility and its deletion results in boosting the flexibility of the ARP module (Fig. S6C). The structural observation is consistent with the functional data that Snf5 deletion adversely affects the interactions of SWI/SNF with nucleosomes and nucleosome remodeling efficiency (Sen et al., 2017). These observations consistently suggest the ARP mobility is important for the SWI/SNF activity.

We visualize four SWI/SNF nucleosome binding states in which the histone binding surface in the foreleg remains largely stable and the mobile ARP module in the thigh constitutes the dynamic interface with extra-nucleosomal DNA (Fig. 2A and 2C). The nucleosome substrate may be firstly captured by the ARP module through the nucleosomal linker DNA, then through the mobility of the ARP, the nucleosome could be delivered to the Snf2 ATPase domain. Previous studies suggest the two lobes of the ATPase domain are structurally dynamic; the ATPase is only active when the two lobes are closely juxtaposed to make the closed ATPase cleft (Chaban et al., 2008; Clapier et al., 2016). It is tempting to propose the substantial conformational changes of the ARP module could play a role in modulating the conformation of the Snf2 ATPase domain, thus controlling the efficiency of SWI/SNF remodeling, which is consistent with previous functional and structural observations on the regulatory role of ARP module (Clapier et al., 2016; Xia et al., 2016). The ARP module may act as a conformational switch to regulate the remodeling activity of the SWI/SNF (Fig. 2D). In short, this study provides the subunit architecture and nucleosome binding for understanding the molecular mechanism of SWI/SNF in catalyzing nucleosome movement.

## Electronic supplementary material

Below is the link to the electronic supplementary material.

Supplementary material 1 (PDF 1290 kb)
