# Supplementary material for: Architecture of SWI/SNF chromatin remodeling complex
Source: Protein Cell. 2018 Mar 15;9(12):1045–9. doi: 10.1007/s13238-018-0524-9 (PMC6251806; doi:10.1007/s13238-018-0524-9)
Supplement: Supplementary file 1 — Supplementary material 1 (PDF 1290 kb) [file 13238_2018_524_MOESM1_ESM.pdf]

Supplementary Information

Architecture of SWI/SNF Chromatin Remodeling Complex

Zhihui Zhang[1], Xuejuan Wang[1, 2✉], Jiyu Xin[1], Zhenrui Ding[1], Sheng Liu[1], Qianglin Fang[3], Na Yang[3], Rui-min Xu[3], Gang Cai[1, 2✉]

[1]Hefei National Laboratory for Physical Sciences at Microscale and School of Life Sciences, University of Science & Technology of China, Hefei, Anhui, China;

[2]CAS Center for Excellence in Molecular Cell Science, Chinese Academy of Sciences, Hefei, 230026, China;

[3]National Laboratory of Biomacromolecules, Institute of Biophysics, Chinese Academy of Sciences, Beijing 100101, China.

✉Correspondence: xuejuan@ustc.edu.cn (X. Wang), gcai@ustc.edu.cn (G. Cai)

MATERIALS AND METHODS

Yeast strains

The budding yeast *Saccharomyces cerevisiae* strains that were used in this study are listed in table S1. A standard one-step polymerase chain reaction (PCR) approach was applied to generate yeast strains harboring individual gene deletions or with modified TAP (Cai et al., 2009) (10 X histidine-TEV-ProteinA) tag at the C terminus of the target protein (Knop et al., 1999). The Arp7, Arp9, and Swi1 subunit deletions are heterozygous deletions and the Snf5 and Swp82 deletion mutant are homozygous deletions. All the modifications were made in the multiple protease-deficient *S. cerevisiae* strain BJ2168 (*MATa leu2 trp1 ura3-52 prb1-1122 pep4-3 prc1-407 gal2)*, except the Snf5 homozygous deletion is in the BY4742 (*MATa SWI3-TAPm snf5Δ:: KanMX leu2Δ0 lys2Δ0 ura3Δ0*) and acquired from the Homozygous Diploid Complete Set (Cat. no. 95401.H1R3, Invitrogen).

Purification of Yeast SWI/SNF and its sub-assemblies

The wild-type or subunit deletion mutant strains were grown in YPD medium to the stationary phase. About 100g cells were harvested, washed and resuspended in extraction buffer (50 mM HEPES (pH 7.6), 300 mM KOAc, 0.5 mM EDTA, 5 mM β-ME, 10% (v/v) glycerol, 0.1% (v/v) NP-40 and protease inhibitors), and a whole-cell extraction was prepared as previously described (Takagi et al., 2005). This whole-cell extract was selectively precipitated in 30–55% ammonium sulfate and resuspended using 1xTEZ buffer (50 mM Tris-HCl (pH 7.5), 1 mM EDTA, 10 mM $ZnCl_2$, 5 mM β-ME and protease inhibitors). After the suspension was clarified using centrifugation, the supernatant was incubated for 2 hours at 4 °C with 1 ml of a 50% slurry of IgG-sepharose resin beads (GE Healthcare) that had been pre-equilibrated with 1xTEZ plus 250 mM ammonium sulfate. After incubation, the beads were washed with 50 ml of 1xTEZ plus 500 mM ammonium sulfate, followed by a second wash with 50 ml of 1xTEZ plus 50 mM ammonium sulfate. After equilibration of the column with 1xTEZ plus 100 mM ammonium sulfate (without protease inhibitors), 100 units of AcTEV

protease (Invitrogen) was added to the resin beads and incubated overnight at 4 °C. The SWI/SNF fraction was then eluted with three column volumes of 1xTEZ plus 100 mM ammonium sulfate and 10% glycerol was added, and the resulting aliquot was snap-frozen in liquid nitrogen and temporarily stored at -80 °C. For the next purification step, the IgG eluate fractions were thawed in ice and applied on a Mono S column (GE Healthcare) in S100 buffer (100mM ammonium sulfate, 50mM Tris pH 7.6, 10% v/v glycerol, 1mM EDTA, 10μM ZnSO4, 0.02% NP-40, 10mM β-ME) and was resolved over a 100–1000 mM ammonium sulfate gradient. The final SWI/SNF fractions eluted at ~400mM ammonium sulfate concentration were flash-frozen in liquid nitrogen.

Assembly of the SWI/SNF-Nucleosome complex

NCP made of bacterially expressed *S. cerevisiae* histones H2A, H2B, H3 and H4 and a 146-bp palindromic DNA fragment derived from human α-satellite DNA was prepared according to a described procedure (Luger et al., 1997). The Mono S peak fraction of the SWI/SNF was incubated with Ni-NTA resin (Quigen) that had been pre-equilibrated with buffer A (20 mM HEPES (pH 7.9), 125 mM NaCl, 2.5 mM $MgCl_2$, 1 mM DTT and 2.6% glycerol), then washed with buffer A. After equilibration of the column with buffer B (20 mM HEPES (pH 7.9), 125 mM NaCl, 2.5 mM $MgCl_2$, 1 mM DTT, 1mM ATPγS, and 2.6% glycerol), 400 pmol nucleosome core particles was added and incubated for 2 hours at 4°C, then was chemical crosslinked with final concentration of 0.04% glutaraldehyde for 5 min under room temperature and quenched the crosslinking with buffer A washing. The SWI/SNF-Nucleosome assembly was eluted with buffer C (20 mM HEPES (pH 7.9), 125 mM NaCl, 2.5 mM $MgCl_2$, 1 mM DTT, 250mM imidazole, and 2.6% glycerol). The resulting aliquot was snap-frozen in liquid nitrogen and temporarily stored at -80 °C.

Negative stain reconstruction

About 3μL sample aliquots (~30 μg protein/ml in 50mM Tris, pH 7.6, 100mM ammonium sulfate, 10% v/v glycerol, 1mM EDTA, 10μM ZnSO4, 0.02% NP-40,

10mM β-ME) was applied to a carbon-coated 300-mesh Cu EM specimen grid freshly glow discharged. The SWI/SNF particles were then preserved by staining with 0.75% (w/w) uranyl formate solution. The particles of the SWI/SNF show a strongly preferred orientation on adsorption to amorphous carbon support films. 3D reconstructions were calculated by using the RCT method (Radermacher, 1988)

We obtained tilted (55°) and untilted image pairs of SWI/SNF particles under low-dose conditions using a Tecnai F20 microscope (FEI) (field emission gun, low-dose conditions, 200-kV accelerating voltage, ~0.6 to 0.8 μm under focus). Images were recorded with a FEI Eagle (4k HS 200kv) CCD camera at 62,000× magnification (1.77 Å per pixel) and two-fold pixel-averaged to 3.54 Å per pixel. Particles were selected using the TiltPicker program (Voss et al., 2009) and montaged them for interactive screening, yielding ~9,000 single particle tilt-pair images. We run iterative alternating rounds of supervised multi-reference alignment and classification as well as reference-free alignment to improve the homogeneity of the image classes. All the 3D reconstructions were calculated with SPIDER (Frank et al., 1996) and SPARX (Hohn et al., 2007). All the 3D structures were displayed using Chimera (Pettersen et al., 2004).

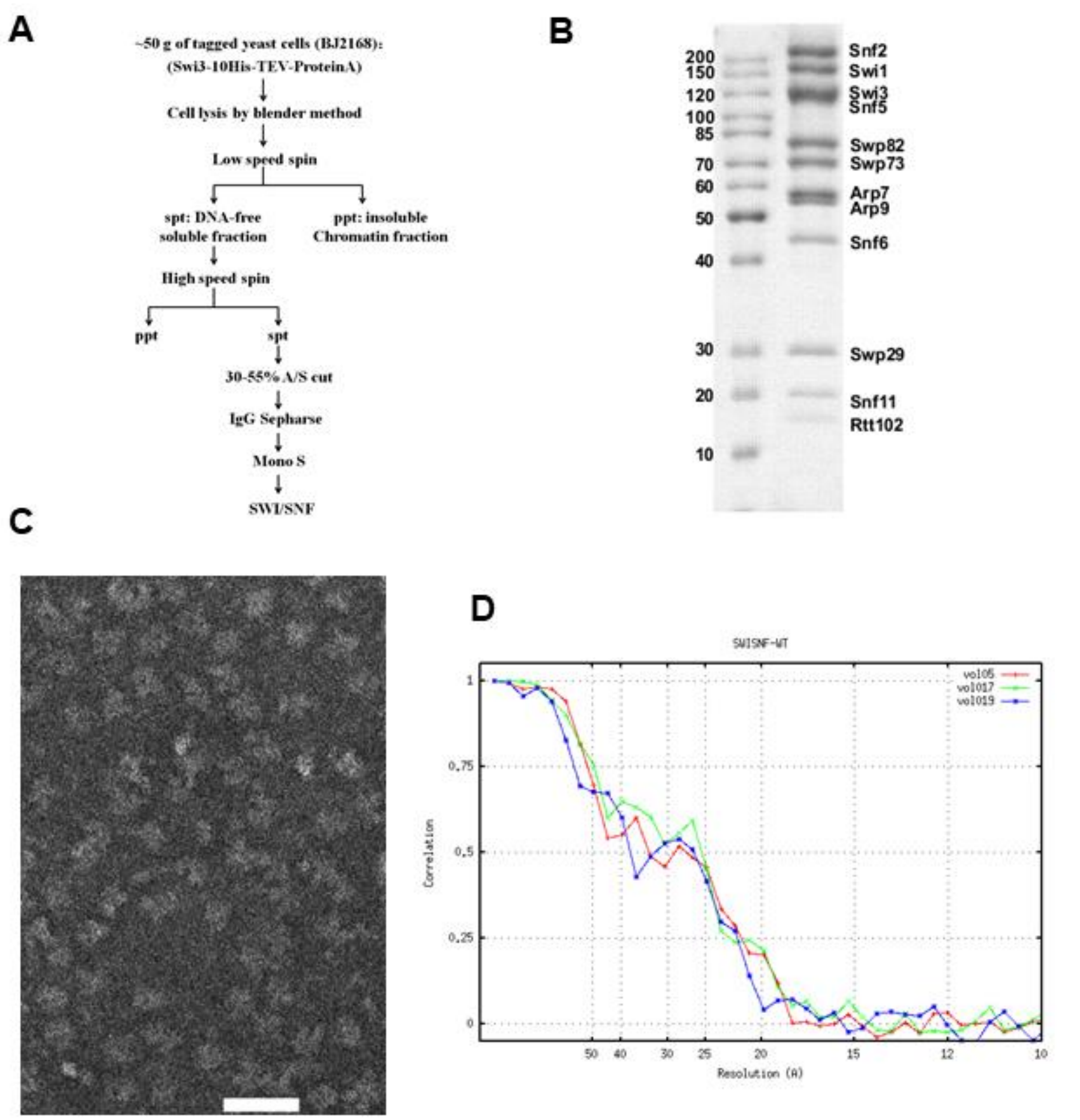

Supplementary Figure 1. EM reconstruction of the intact SWI/SNF complex.

(A) Scheme for affinity purification of SWI/SNF complex from the whole cell extraction of tagged *S. cerevisiae* cells. (Abbreviations: spt, supernatant; ppt, precipitation.) (B) The SDS-PAGE analysis of the 12-subunit SWI/SNF complex. (C) A micrograph showing single SWI/SNF particles preserved under stain. Scale bar, 50 nm. (D) Fourier Shell Correlation (FSC) curves for the RCT reconstructions. Using a FSC = 0.5 criterion, the resolution of the maps are estimated to be ~25~38 Å.

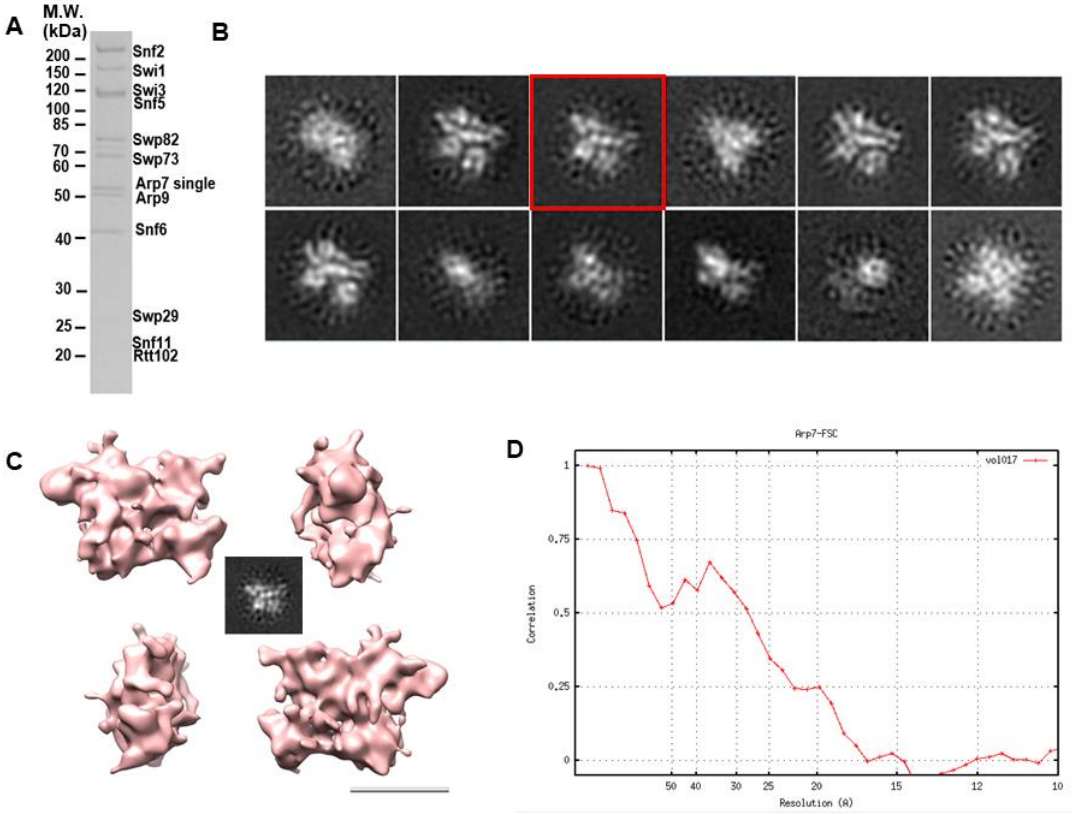

Supplementary Figure 2. EM reconstruction of the SWI/SNF subcomplex purified from Arp7 deletion strain.

(A) Ruby-stain SDS-PAGE analysis of the subcomplex endogenously purified from the *S.cerevisiae* Arp7Δ strain. (B) 2D averages of the SWI/SNF subcomplex from the Arp7Δ strain. Red square represents the class used in further analysis. (C) 2D and 3D EM analysis of the subcomplex from the Arp7Δ strain. Scale bar, 100 Å. (D) Fourier Shell Correlation (FSC) curve for the RCT reconstruction of the Arp7Δ subcomplex. Using a FSC = 0.5 criterion, the resolution of the map is estimated to be ~28 Å.

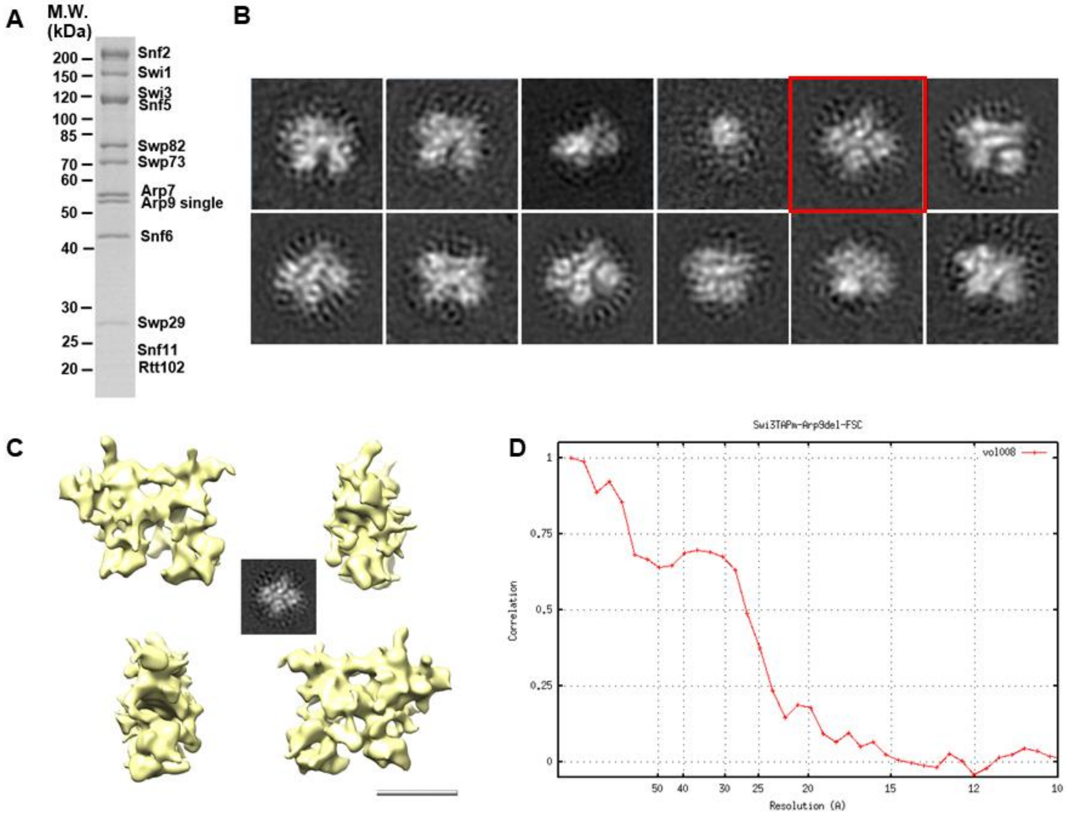

Supplementary Figure 3. EM reconstruction of the SWI/SNF subcomplex purified from Arp9 deletion strain.

(A) Ruby-stain SDS-PAGE analysis of the subcomplex endogenously purified from the *S.cerevisiae* Arp9Δ strain. (B) 2D averages of the SWI/SNF subcomplex from the Arp9Δ strain. Red square represents the class used in further analysis. (C) 2D and 3D EM analysis of the subcomplex from the Arp9Δ strain. Scale bar, 100 Å. (D) Fourier Shell Correlation (FSC) curve for the RCT reconstruction of the Arp9Δ subcomplex. Using a FSC = 0.5 criterion, the resolution of the map is estimated to be ~24 Å.

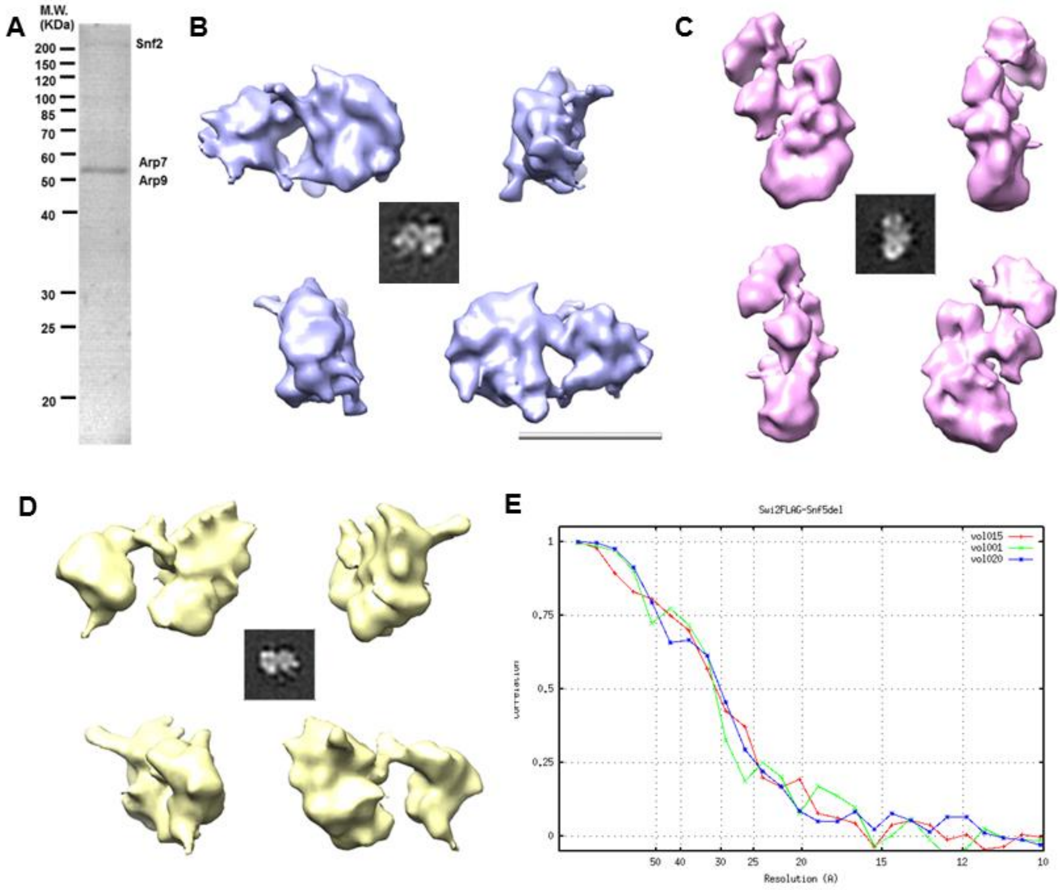

Supplementary Figure 4. EM reconstruction of the catalytic core subcomplex purified from the Snf2-FLAG/Snf5Δ strain.

(A) Ruby-stain SDS-PAGE analysis of the catalytic core subcomplex containing Snf2, Arp7 and Arp9. (B, C, D) 2D and 3D EM analysis of the three conformations of the catalytic core subcomplex. Scale bar, 100 Å. (E) Fourier Shell Correlation (FSC) curve for the RCT reconstruction. Using a FSC = 0.5 criterion, the resolution of the maps are estimated to be ~30 Å.

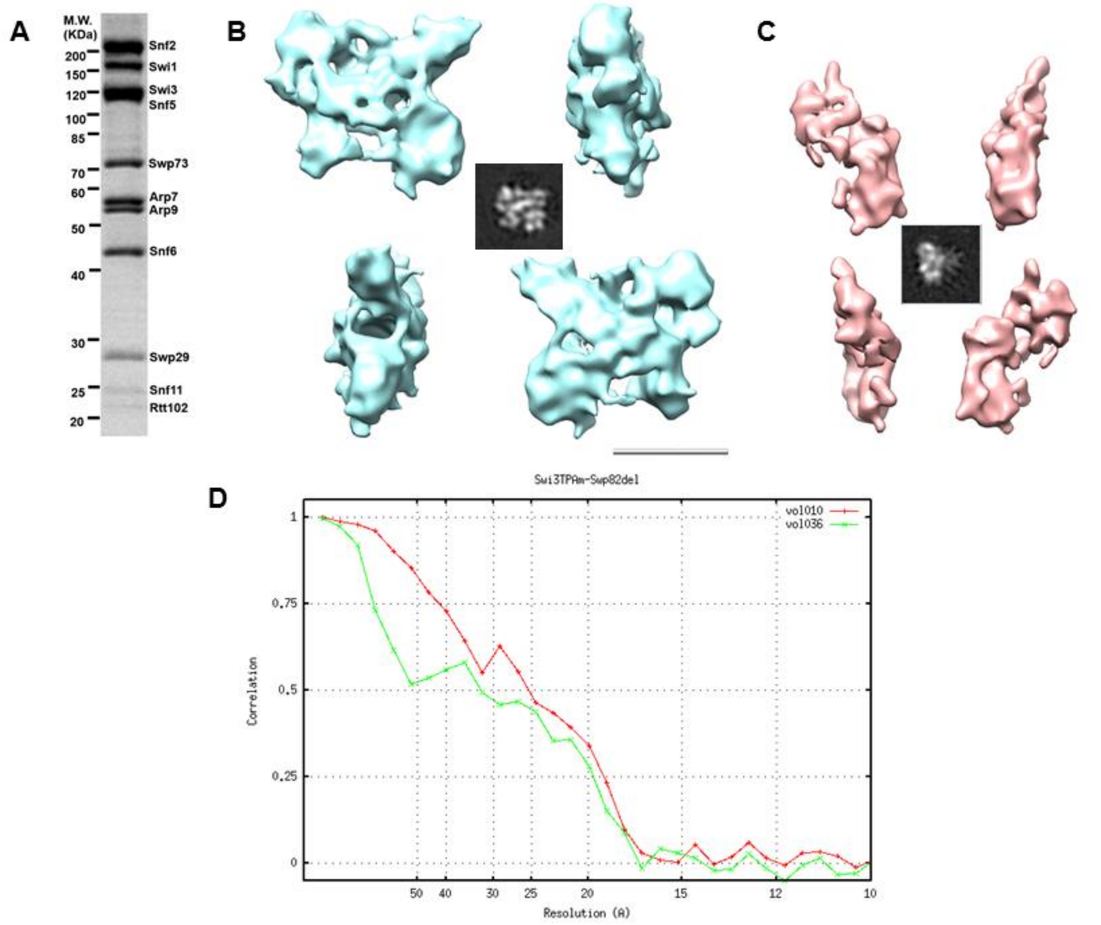

Supplementary Figure 5. EM reconstruction of the SWI/SNF subcomplex purified from *S. cerevisiae* Swp82Δ strain.

(A) Ruby-stain SDS-PAGE analysis of the subcomplex endogenously purified from the *S.cerevisiae* Swp82Δ strain. (B, C) 2D and 3D EM analysis of the two subcomplex from the Swp82Δ strain. Scale bar, 100 Å. (D) Fourier Shell Correlation (FSC) curve for the RCT reconstruction of the Swp82Δ subcomplex. Using a FSC = 0.5 criterion, the resolution of the maps are estimated to be ~25~32 Å.

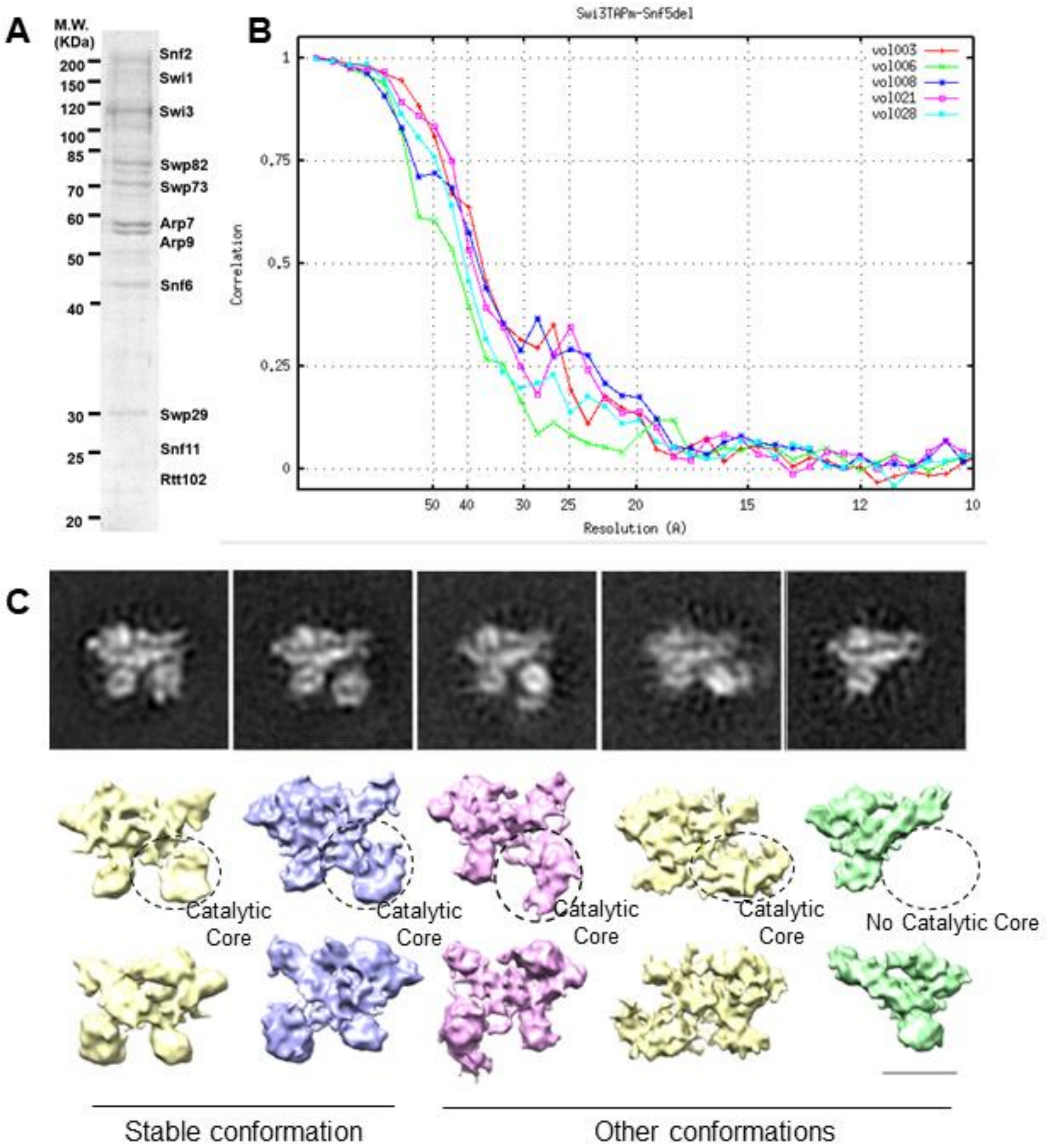

Supplementary Figure 6. EM reconstruction of the subcomplex purified from *S. cerevisiae* Snf5Δ strain.

(A) Ruby-stain SDS-PAGE analysis of the subcomplex endogenously purified from the *S.cerevisiae* Snf5Δ strain. (B) Fourier Shell Correlation (FSC) curve for the RCT reconstruction of the Snf5Δ subcomplex. Using a FSC = 0.5 criterion, the resolutions of the maps are estimated to be ~37~42 Å. (C) 2D and 3D EM analysis of the subcomplex from the Snf5Δ strain. At least six different conformations were identified through reference-free alignment and classification of EM images. Distinct structures of the Snf5 deletion mutant. The mobility of the catalytic core is substantially boosted in the absence of the Snf5 subunit, which even could be fully detached. Scale bar, 100 Å.

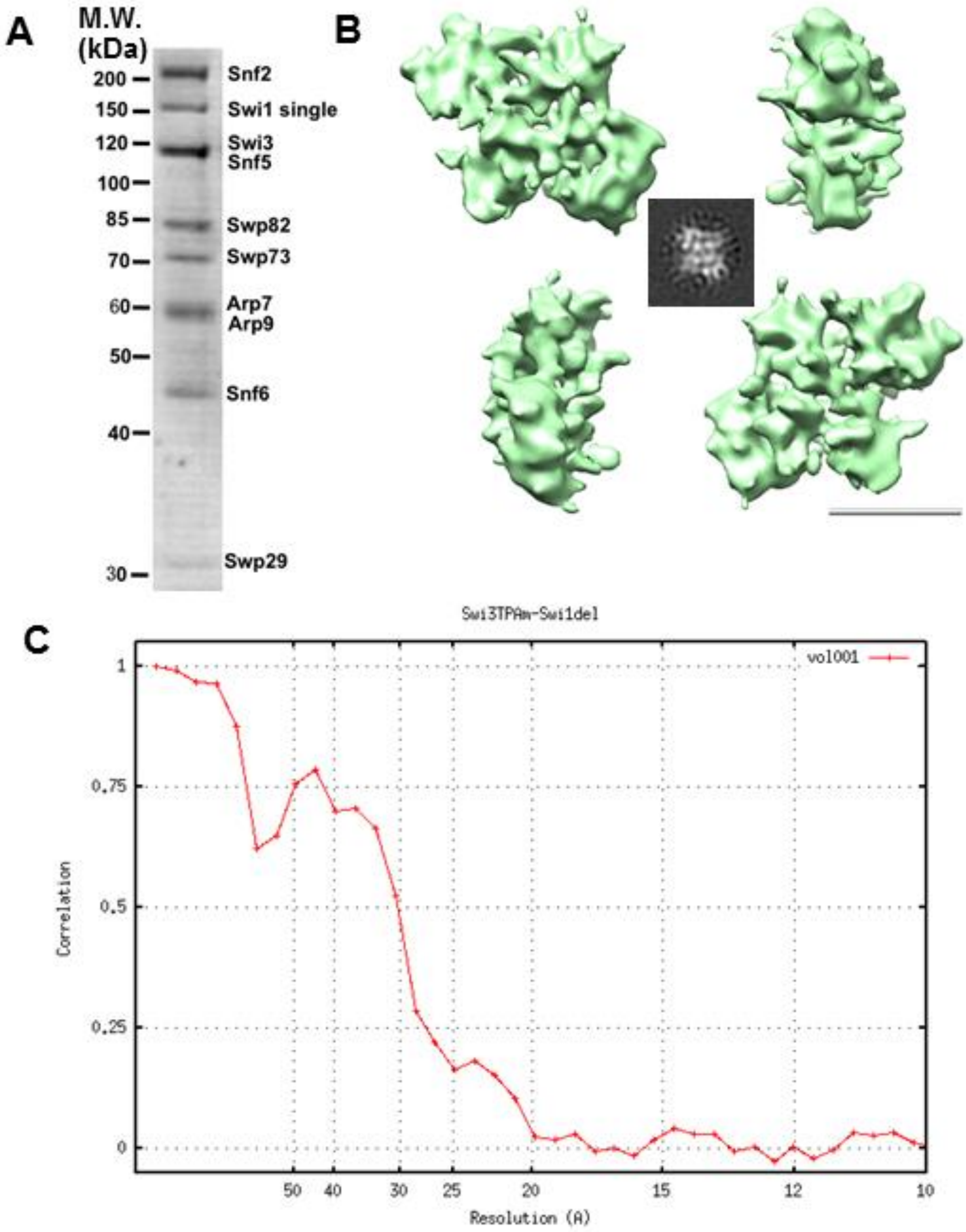

Supplementary Figure 7. EM reconstruction of the subcomplex purified from *S. cerevisiae* Swi1 heterozygous deletion strain.

(A) Ruby-stain SDS-PAGE analysis of the subcomplex endogenously purified from the *S.cerevisiae* Swi1Δ strain. (B) 2D and 3D EM analysis of the subcomplex from the Swi1Δ strain. Scale bar, 100 Å. (C) Fourier Shell Correlation (FSC) curve for the RCT reconstruction of the Swi1Δ subcomplex. Using a FSC = 0.5 criterion, the resolution of the map is estimated to be ~30 Å.

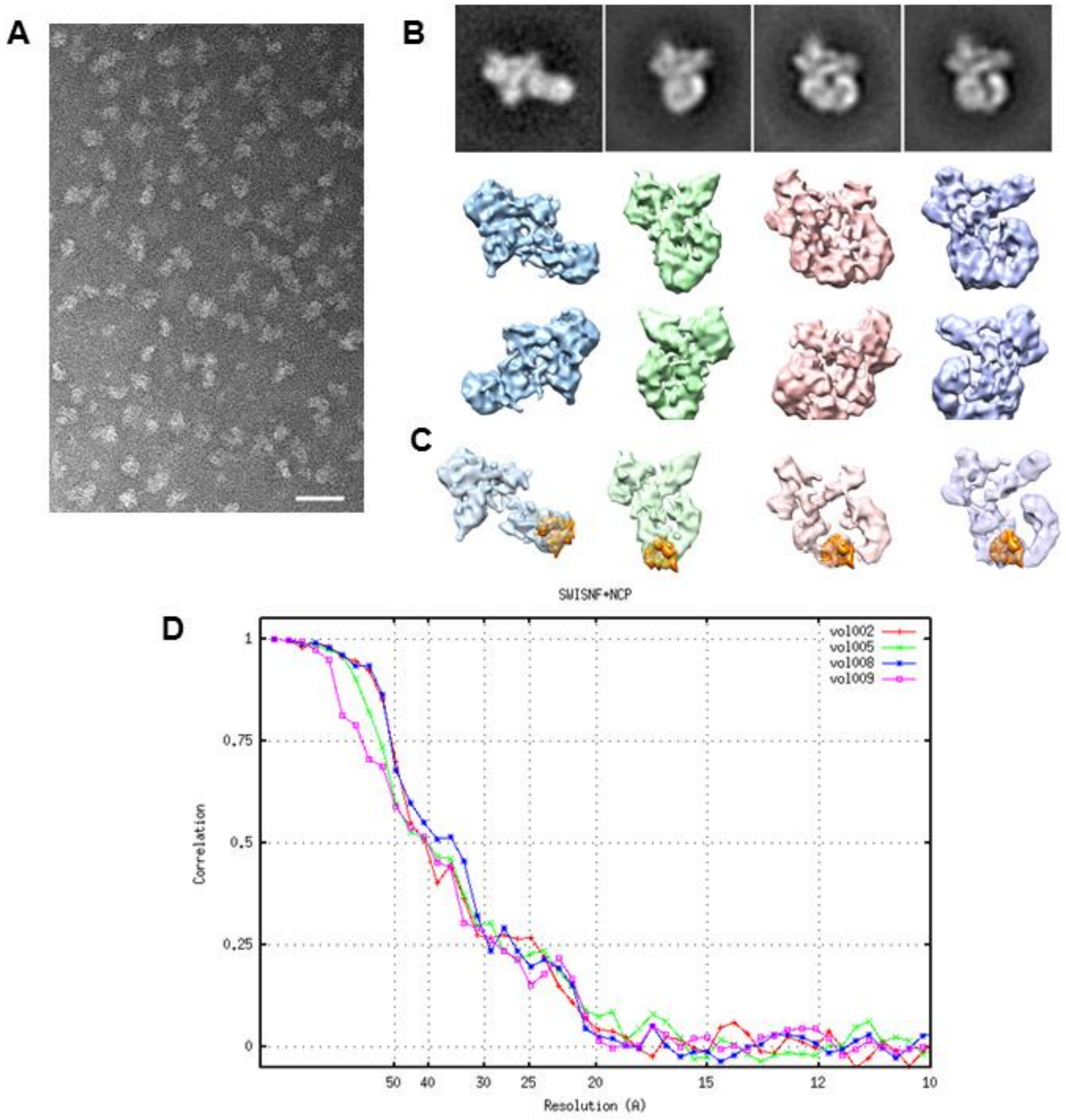

Supplementary Figure 8. EM reconstructions of the four nucleosome binding states of the SWI/SNF.

(A) A micrograph showing single particles of the SWI/SNF binding a nucleosome preserved under stain. Scale bar, 50 nm. (B) 2D and 3D EM analysis of the four states. (C) 3D maps of the four nucleosome binding states showing at the lower density threshold with the bound nucleosomes highlighted by solid orange densities. (D) Fourier Shell Correlation (FSC) curves for the RCT reconstruction of the complex. Using a FSC = 0.5 criterion, the resolution of the maps are estimated to be ~34~40 Å.

Supplementary Table 1

Yeast strains used in this study.

Strains	Genotype	Genetic Background
SWI3-TAPm	*MATa SWI3-TAPm leu2 ura3-52 prb1-1122 pep4-3 prc1-407 gal2*	BJ2168
SWI3-TAPm-ARP9Δ*	*MATa SWI3-TAPm arp9Δ:: KanMX leu2 ura3-52 prb1-1122 pep4-3 prc1-407 gal2*	BJ2168
SWI3-TAPm-ARP7Δ*	*MATa SWI3-TAPm arp7Δ:: KanMX leu2 ura3-52 prb1-1122 pep4-3 prc1-407 gal2*	BJ2168
SWI3-TAPm-SWP82Δ	*MATa SWI3-TAPm swp82Δ:: KanMX leu2 ura3-52 prb1-1122 pep4-3 prc1-407 gal2*	BJ2168
SWI3-TAPm-SWI1Δ*	*MATa SWI3-TAPm swi1Δ:: KanMX leu2 ura3-52 prb1-1122 pep4-3 prc1-407 gal2*	BJ2168
SWI3-TAPm-SNF5Δ	*MATa SWI3-TAPm snf5Δ:: KanMX leu2Δ0 lys2Δ0 ura3Δ0*	BY4742
SNF2-FLAG-SNF5Δ	*MATa SNF2-FLAG snf5Δ:: KanMX leu2Δ0 lys2Δ0 ura3Δ0*	BY4742

Note: * represents heterozygous deletions

References

Cai, G., Imasaki, T., Takagi, Y., and Asturias, F.J. (2009). Mediator structural conservation and implications for the regulation mechanism. Structure 17, 559-567.

Frank, J., Radermacher, M., Penczek, P., Zhu, J., Li, Y., Ladjadj, M., and Leith, A. (1996). SPIDER and WEB: processing and visualization of images in 3D electron microscopy and related fields. J Struct Biol 116, 190-199.

Hohn, M., Tang, G., Goodyear, G., Baldwin, P.R., Huang, Z., Penczek, P.A., Yang, C., Glaeser, R.M., Adams, P.D., and Ludtke, S.J. (2007). SPARX, a new environment for Cryo-EM image processing. J Struct Biol 157, 47-55.

Knop, M., Siegers, K., Pereira, G., Zachariae, W., Winsor, B., Nasmyth, K., and Schiebel, E. (1999). Epitope tagging of yeast genes using a PCR-based strategy: more tags and improved practical routines. Yeast 15, 963-972.

Luger, K., Rechsteiner, T.J., Flaus, A.J., Waye, M.M., and Richmond, T.J. (1997). Characterization of nucleosome core particles containing histone proteins made in bacteria. J Mol Biol 272, 301-311.

Pettersen, E.F., Goddard, T.D., Huang, C.C., Couch, G.S., Greenblatt, D.M., Meng, E.C., and Ferrin, T.E. (2004). UCSF Chimera--a visualization system for exploratory research and analysis. J Comput Chem 25, 1605-1612.

Radermacher, M. (1988). Three-dimensional reconstruction of single particles from random and nonrandom tilt series. J Electron Microsc Tech 9, 359-394.

Takagi, Y., Chadick, J.Z., Davis, J.A., and Asturias, F.J. (2005). Preponderance of free mediator in the yeast Saccharomyces cerevisiae. J Biol Chem 280, 31200-31207.

Voss, N.R., Yoshioka, C.K., Radermacher, M., Potter, C.S., and Carragher, B. (2009). DoG Picker and TiltPicker: software tools to facilitate particle selection in single particle electron microscopy. J Struct Biol 166, 205-213.
